# Methylenetetrahydrofolate Reductase C677T and A1298C Polymorphism in Iranian Women With Idiopathic Recurrent Pregnancy Losses

**DOI:** 10.5812/ircmj.16763

**Published:** 2014-07-05

**Authors:** Elham Yousefian, Mohammad Taghi Kardi, Azra Allahveisi

**Affiliations:** 1 Department of Midwifery, Islamic Azad University Falavarjan Branch, Isfahan, IR Iran; 2 Department of Biology, University of Isfahan, Isfahan, IR Iran; 3 Department of Anatomy, Faculty of Medicine, Kurdistan University of Medical Sciences, Sannandaj, IR Iran

**Keywords:** Methylenetetrahydrofolate Reductase, Thrombophilia, Pregnancy

## Abstract

**Background:**

Recurrent pregnancy loss (RPL) is a serious problem for pregnancy. There is evidence that vascular complications play a principal role in RPL. Methylenetetrahydrofolate reductase (MTHFR) is a key enzyme in folate metabolism. Polymorphisms (C677T, A1298C) of MTHFR gene are associated with decreased MTHFR activity.

**Objectives:**

The aim of this study was to determine the association between MTHFR polymorphisms (C677T and A1298C) and recurrent pregnancy loss (RPL) in Iranian women.

**Materials and Methods:**

In this case-control study, blood samples were obtained from patients who had three or more consecutive pregnancy losses before the 22^nd^ week of pregnancy (n = 204). The control group consisted of 116 age-matched women with at least one alive child and without any history of pregnancy loss or other gestational complications (n = 116). Following DNA extraction, samples were tested for MTHFR C677T and A1298C polymorphisms using the reverse hybridization method.

**Results:**

The prevalence of 677TT mutation was 8.8% (18/204) in the patient group and 8.6% (10/116) in the control group (P = 0.434). The prevalence of 1298CC mutation was 12.3 % (25/204) in the patient group and 8% (9/116) in the control group (P = 0.155). Investigation of the distributions of various genotypes of MTHFR C677T and A1298C did not indicate a significant difference between patients with RPL and healthy control subjects.

**Conclusions:**

The results suggest that MTHFR mutations might not be associated with RPL in the examined population.

## 1. Background

Recurrent pregnancy loss (RPL) is classically defined as three (two in some studies) or more spontaneous fetal losses before the 22^nd^ week of pregnancy. It is a serious condition affecting 5% of pregnant women and it has devastating social and medical consequences (1). The placenta should develop and function well because it is a crucial factor in a successful pregnancy. Thrombosis that is a result of microthrombi in the placental vascular bed may impair placental perfusion. Microthrombi creates multiple infracts, which cause complications such as placental abruption, intrauterine growth retardation (IUGR), and early onset of preeclampsia (2). Since placental vascular lesions have a thrombotic nature and there is an association between thrombotic risk and thrombophilia; it can be suggested that hereditary thrombophilia may result in severe pregnancy complications. Normal balance of procoagulant and anticoagulant mechanisms influence adequate placental circulation; therefore, inherited thrombophilia can be associated with fetal loss. The methylenetetrahydrofolate reductase (MTHFR) gene is composed of 11 exons, which are located on the short arm of chromosome 1 (1p36.3) (3). The MTHFR enzyme catalyzes the reduction of 5, 10-methylenetetrahydrofolate to 5-methyltetrahydrofolate (4). The MTHFR enzyme is an important regulatory enzyme involved in folate metabolism and is a critical factor in DNA methylation (5, 6). There are two common polymorphisms in the MTHFR gene (7, 8). MTHFR C677T (rs1801133) and MTHFR A1298C (rs1801131). The C677T (Ala222Val) polymorphism results in alanine-to-valine substitution at codon 222 (9). The A1298C (Glu429Ala) results in glutamate-to-alanine substitution at codon 429. The MTHFR C677T variant results in a thermo labile protein with enzymatic activity that is decreased by 35% in the heterozygote state (CT genotype) and by 70% in the homozygote state (TT genotype) (9, 10). As far as we know, the A1298C polymorphism has not been completely studied before; also it has been suggested that people with 1298CC genotype have roughly 60% of the enzyme activity found in people who have the common AA genotype (11).

## 2. Objectives

The present study aimed to test the association between MTHFR polymorphisms (C677T, A1298C) and RPL in Iranian women. The frequencies of these mutations were statistically compared between cases with RPL and a control group of healthy women to examine whether significant differences exist between the two groups regarding polymorphisms frequencies.

## 3. Materials and Methods

### 3.1. Subjects

Ethical approval for this study was obtained from the Ethics Committee of Isfahan University of Medical Sciences (code. no. 492024). All participants signed an informed consent form. This case-control study was performed on 204 women (25 to 38 years old) with RPL, who were referred to the Dr Baradaran laboratory by specialists of the Isfahan province (Iran) between 2009 and 2013. On the other hand, 116 women were also studied as the control group. The eligibility criterion was the existence of three or more consecutive pregnancy losses before the 22^nd^ week of pregnancy, regardless of a previous live birth. Exclusion criteria were induced abortions, infection, systemic diseases and uterine structural abnormalities. The control group consisted of 116 age-matched women with at least one alive child and without a history of pregnancy loss or other gestational complications. Both patients and control subjects were born in the central areas of Iran and were living in either the Isfahan province or a nearby region. A blood sample was collected from each woman and placed in EDTA for DNA extraction and molecular analysis (Table 1).

**Table 1.** Characteristics of Patients and Controls^a^

	Patients	Controls	P Value
**Number of Abortion**	2.34 ± 0.48	0	
**Age, y**	29.7 ± 3.4	30.4 ± 3.2	0.17
**BMI, kg/m** ^**2**^	24.2 ± 2.5	25.4 ± 2.7	0.23

^a^ Abbreviation: BMI, body mass index.

### 3.2. Molecular Diagnosis

DNA was extracted from whole blood specimens, using a commercially available QIAamp DNA mini kit (Code No. 513041; Qiagen, Germany). PCR constituents made up a final volume of 50 μL, consisting of of 35 μL MTHFR mix, 5 μL of 10x polymerase buffer, 1.25 μL of MgCl_2_ (concentration 100 mM), 0.2 μL Taq DNA polymerase, 2 μL of DNA (150-200 ng) and 6.55 μL nuclease free water. The PCR amplification included the following steps; step 1 initial denaturation (1 cycle for 5 minutes at 95°C), step 2 denaturation (10 cycles for 30 seconds at 95°C and 2 minutes at 60°C), step 3 annealing (22 cycles for 10 seconds at 95°C, 30 seconds at 55°C and 30 seconds at 72°C), and step 4 elongation (1 cycle for 8 minutes at 72°C followed by holding at 4°C at the end of the reaction). After performing the PCR reaction, the amplified gene fragments were characterized by sequence-specific oligonucleotide probes in a hybridization reaction (SSOP-PCR). The gene fragments were immobilized on nitrocellulose according to the manufacturer’s instructions (reverse hybridization kits obtained from GenID GnbH Ebinger Straberg). The procedure was as follows: incubation trays were prepared for the samples and controls. Next, 20 µL of denaturing reagent was added using a pipette to the marked wells. Then, 20 µL of amplicon from the PCR was added to each drop of denaturing reagent. These mixtures were mixed well and incubated at room temperature for 5 minutes. Each nitrocellulose strip had gene probes for the wild type and mutated sequence of the tested genes and various control zones. The strips were placed in the incubation tray. Next, 1 mL of hybridization buffer was added to the mixture. In the next step, the strips were incubated and slightly shaken for 30 minutes at 47°C in a water bath. They were then washed twice for 1 minute with 1 mL diluted rinse. Then, 1 µL of conjugate was added to all strips. While shaken slightly, they were incubated for 30 minutes at room temperature. Then, the strips were washed 3 times for 1 minute with diluted rinse and 1 mL of substrate was added to each strip, followed by incubation for 10-20 minutes. The reaction was interrupted by washing the strips twice with distilled water. During the hybridization step, the denatured amplified DNA bound to the gene probes attached to the strips. A highly specific washing procedure ensured that the hybrids would survive, only if the probe sequence was 100% complementary to that of the amplified DNA. This complex can be detected by a color reaction of BCIP/NBT (5-bromo-4-chloro-3'-indolyphosphate and nitro-blue tetrazolium) at the alkaline phosphatase. The banding pattern of the strips could be analyzed using a kit-specific evaluation sheet.

### 3.3. Statistical Analysis

Statistical data analysis was performed using SPSS version 20 (SPSS Inc., Chicago, IL, USA). The mutation frequencies in MTHFR between case and the control group were analyzed using the Pearson’s chi-square test as well as the odd ratios (ORs) with 95% confidence intervals (95% CIs). P values < 0.05 were considered significant.

## 4. Results

The prevalence of MTHFR mutation was determined for 204 women with RPL of unexplained etiology and 116 women who had a history of uncomplicated pregnancies. The frequencies of MTHFR 677TT among cases and controls were 8.8% and 8.6%, respectively. Overall, distribution of the various genotypes of MTHFR C677T did not differ significantly among patients with recurrent unexplained spontaneous abortions and healthy control subjects (P = 0.434). The results are summarized in Table 2.

**Table 2.** Distribution of Methylenetetrahydrofolate Reductase C677T and A1298C Genotypes^a^

MTHFR	Genotype	Case, No. (%)	Control, No. (%)	OR	CI 95%
**C677T**					
	CC	96 (47.1)	63 (54.3)		
	CT	90 (44.1)	43 (37.1)	1.374	0.848-2.226
	TT	18 (8.8)	10 (8.6)	1.181	0.512-2.725
**A1298C**					
	AA	98 (48.0)	68 (58)		
	AC	81 (39.7)	39 (33.6)	1.441	0.882-2.356
	CC	25 (12.3)	9 (7.8)	1.927	0.847-4.386

^a^ Abbreviations: MTHFR, methylenetetrahydrofolate reductase; OR, odd ratio.

The frequencies of MTHFR 1298CC were similar (12.3% and 7.8%) among women with RPL and healthy controls. 1298CC was more frequent in RPL women; however, this difference was not significant (P = 0.155). The results are summarized in Table 2. The prevalence of heterozygous C677T and A1298C was similar. The distribution of MTHFR, C677T and A1298C genotypes did not differ significantly between patients with recurrent unexplained spontaneous abortions and healthy control subjects (Table 2).

## 5. Discussion

The results suggest no statistically significant difference in MTHFR mutations between patients with RPL and control patients. Bae et al. indicated that MTHFR mutations in Korean women was not a significant risk factor for idiopathic RSA (12). Abu-Asab et al. did not find a significant association between MTHFR and RPL in either the first or second trimester (1). Nelen et al. performed a meta-analysis of 10 case-control studies to assess the risks of hyperhomocysteinemia and MTHFR C677T mutations in recurrent early pregnancy loss and found that elevated blood homocysteine and the variant homozygous genotype 677TT of MTHFR 677 CT polymorphism are risk factors for recurrent early pregnancy loss (13). According to a more recent meta-analysis of 26 case-control studies done by Ren and Wang, the genotypes of MTHFR C677T had strong significant associations with RPL; it was concluded that there is a positive association between the examined genotypes and RPL. However, the conclusion of this meta-analysis was based on only five Chinese studies that exhibited very strong correlations between MTHFR C677T polymorphism and RPL, although none of the other 21 studies showed any association (14). MTHFR mutation results in reduced activity and enhanced thermolability of the enzyme and subsequent elevation of homocysteine levels. Clinically, hyperhomocysteinemia caused by the mutation, has been associated with coronary artery disease, venous thrombosis and complications of pregnancy (15). Thus, it was of interest to study MTHFR in women with RPL because placental infarcts have been associated with RPL (16). However, MTHFR was underrepresented in the RPL group, which was in agreement with other reports (17, 18). This might be due to the use of folic acid among the pregnant women, especially during the first trimester. Folate level plays a significant role in regulating homocysteine in individuals homozygous for MTHFR (18). Therefore, it was reasonable to assume that folic acid consumption may effectively reduce the adverse effects of MTHFR and decrease the risk of RPL in women who were homozygous for this mutation (1). In agreement with the other results (1, 19), the current study did not find any correlation between the prevalence of homozygosis or heterozygosis for the MTHFR C677T and A1298C mutation between patients with RPL and healthy women. These mutations might not contribute to early RPL in the examined population. The mutations may result in second and third trimester fetal loss. Large-scale studies that would exclude patients with genetically aberrant embryos seem necessary to confirm these results.
